# Supplementary material for: Moral foundations theory, political identity, and the depiction of morality in children’s movies
Source: PLoS One. 2021 Mar 26;16(3):e0248928. doi: 10.1371/journal.pone.0248928 (PMC7996984; doi:10.1371/journal.pone.0248928)
Supplement: S1 Appendix — (DOCX) [file pone.0248928.s001.docx]

# **S1 Appendix (Movies Included in Study 1)**

Cinderella (1950)

Treasure Island (1950)

Alice in Wonderland (1951)

Peter Pan (1953)

Lady and the Tramp (1955)

Old Yeller (1957)

Sleeping Beauty (1959)

101 Dalmatians (1961)

In Search of the Castaways (1962)

Mary Poppins (1964)

The Sound of Music (1965)

The Jungle Book (1967)

The Aristocats (1970)

Fiddler on the Roof (1970)

Bedknobs and Broomsticks (1971)

The Rescuers (1977)

Benji (1974)

Robin Hood (1973)

The Muppet Movie (1979)

The Fox and the Hound (1981)

E.T the Extra-Terrestrial (1982)

Pee-wee's Big Adventure (1985)

Short Circuit (1986)

The Great Mouse Detective (1986)

Home Alone (1990)

Beauty and the Beast (1991)

Aladdin (1992)

The Lion King (1994)

Toy Story (1995)

The Santa Clause (1994)

Shrek (2001)

Harry Potter and the Sorcerer’s Stone (2001)

Monsters, Inc. (2001)

Finding Nemo (2003)

Shrek 2 (2004)

Shrek the Third (2007)

Up (2009)

Toy Story 3 (2010)

Alice in Wonderland (2010)

Frozen (2013)

Finding Dory (2016)

Beauty and the Beast (2017)
