# Supplementary material for: Moral foundations theory, political identity, and the depiction of morality in children’s movies
Source: PLoS One. 2021 Mar 26;16(3):e0248928. doi: 10.1371/journal.pone.0248928 (PMC7996984; doi:10.1371/journal.pone.0248928)
Supplement: S2 Appendix — (DOCX) [file pone.0248928.s002.docx]

# **S2 Appendix (Moral Foundations Coding Scheme)**

This evaluation is an adaptation of the Moral Foundations Questionnaire 30. It should be completed for each central character. A character is an agent with human-like mental processes. Central characters are those who appear in at least 20% of scenes. In each of these scenes, central characters appear in at least 3 camera cuts and speak two or more sentences (unless the character is unable to speak, in which case nonverbal communication is expected regularly). A scene is “a portion of the movie in which the narrative and action form a complete whole” [48].

For a given character, rate each moral foundation on a scale from one to five. Evaluations should be based on what the producers intend for the viewer to take away. If there is not enough information about the character to evaluate a foundation, input “.” on the spreadsheet. If a character is inconsistent in displaying a trait (for example the character is respectful of some authority figures but not others), the character should score a three. If the character overwhelmingly acts in a way consistent with a given value, but acts in a way inconsistent with that value in a few instances, then the character should score a 4 (for example, the character expresses care for all other characters except for one). If a character’s moral values undergo a dramatic change over the course of the movie, the score should be based on the values at the end of the movie.

**Care/Harm**

Score:**____**

*To score a 5, the character:*

- Expresses empathy and compassion
- Nurtures and protects the vulnerable or suffering
- Despises cruelty and exploitation
- Is sensitive to the emotions of others

*To score a 3, the character:*

- Expresses aspects of both 1 and 5

*To score a 1, the character:*

- Intentionally harms others*
- Is cruel or selfish
- Shows indifference to the suffering of others

*To score an unclear, the character does not demonstrate any of the qualities listed above.*

*Violence committed out of self-defense or out of defense of a vulnerable character does not

give the perpetrator a lower score on the care criteria.

**Fairness**

Score:____

*To score a 5, the character:*

- Values fairness, justice and revenge
- Does not cheat when given the opportunity, and is angry at the cheating of others
- Values equality or proportionality

*To score a 3, the character:*

- Expresses aspects of both 1 and 5

*To score a 1, the character:*

- Tries to get more than he/she deserves
- Intentionally profits from another character’s loss
- Steals or cheats

*To score an unclear, the character does not demonstrate any of the qualities listed above.*

**Loyalty*** (to groups that are central to the plot; this might include family, friends, community, or country)

Score:____

*To score a 5, the character:*

- Is willing to make sacrifices for the group
- Values conformity to group norms
- Actively tries to benefit the group
- Has pride in group’s history
- Hates traitors
- Holds the belief that “It is more important to be a team player than to express oneself.”

*To score a 3, the character:*

- Expresses aspects of both 1 and 5

*To score a 1, the character:*

- Is a traitor or betrays the group
- Is unwilling to make sacrifices for the group
- Breaks norms and traditions
- Is critical of the group

*To score an unclear, the character does not demonstrate any of the qualities listed above.*

*If a character is loyal to a friend/family member, but is not loyal to the group or breaks group norms, then that character receives a 3 for loyalty.

**Authority** (authority figures could include parents, group leaders, politicians, teachers, God)

Score:____

*To score a 5, the character:*

- Values obedience to authority
- Shows respect to authority
- Holds the belief that children should be respectful of parents, teachers, and other figures of authority

*To score a 3, the character:*

- Expresses aspects of both 1 and 5

*To score a 1, the character:*

- Resents or is critical of authority
- Values rebellion or rebels against authority

*To score an unclear, the character does not demonstrate any of the qualities listed above.*

**Sanctity**:

Score:____

*To score a 5, the character:*

- Values cleanliness, purity, or chastity
- Judges others for being disgusting or impure
- Holds the belief that “people should not do things that are disgusting, even if no one is harmed”
- Holds the belief that “some acts are wrong on the grounds that they are unnatural.”

*To score a 3, the character:*

- Expresses aspects of both 1 and 5

*To score a 1, the character:*

- Acts in a disgusting or indecent way
- Is irreverent to sacred values* or objects (eg. stepping on the flag, swearing in a church)

*To score an unclear, the character does not demonstrate any of the qualities listed above.*

*A sacred value is “any value that a moral community implicitly or explicitly treats as possessing infinite or transcendental significance that precludes comparisons, trade-offs, or indeed any other mingling with bounded or secular values” [49, p. 853].
