# Supplementary material for: Moral foundations theory, political identity, and the depiction of morality in children’s movies
Source: PLoS One. 2021 Mar 26;16(3):e0248928. doi: 10.1371/journal.pone.0248928 (PMC7996984; doi:10.1371/journal.pone.0248928)
Supplement: S3 Appendix — (DOCX) [file pone.0248928.s003.docx]

# **S3 Appendix (Additional Characteristics Coding Scheme)**

*This evaluation should be completed for each central character. Evaluations should be based on what the producers* intend *for the viewer to take away. If there is not enough information about the character to evaluate this foundation, input “.” The defaults for “attractiveness” and “weight” are average; 2. These should be evaluated in comparison to others of the same species and age. All monsters are of one species, and all toys are of one species.*

**Gender**: ____

Male: 1

Female: 2

**Language/accent**:____

Not standard English; voice sounds significantly different from other characters: 1

If yes, what type? ________

Standard English: 2

Character does not speak: 3

**Estimated Age in Human Years**:____

6 or younger: 1

7-12: 2

13-17: 3

18-50: 4

51-64: 5

65 or older: 6

Multiple/age changes throughout movie: 7

**Attractiveness**:____

Unattractive: 1

“ugly, unappealing, or below-average in looks”

Ordinary: 2

“average or normal looking”

Attractive or cute: 3

“good-looking, pretty, handsome, or above-average in attractiveness,” or unusually cute [25]

Changes from unattractive to attractive: 4

Changes from attractive to unattractive: 5

**Weight classification** (Compared to others of the same age and species): ____

Overweight or obese: 1

Average weight: 2

Underweight: 3

**Violence**:____

Engages in significant physical violence*: 1

Does not engage in significant physical violence: 2

*Violence is behavior involving physical force intended to hurt, damage, or kill someone or something

**Heroic or villainous**:____

Character is depicted as a “bad guy;” behavior is meant to seem despicable or evil. The goals of

the villain are in opposition to the goals of the hero; the character is “not on your side”: 1

Character is depicted as a “good guy;” behavior is meant to be emulated. The viewer is meant to

cheer on and root for this character; the character is “on your side”: 2

Neither; the character is portrayed sometimes as good and sometimes as bad: 3

Character changes from good to bad: 4

Character changes from bad to good: 5

**Outcome for this character by end of movie**:

Undesirable outcome; the character does not achieve his/her goals, or experiences unhappiness or unpleasant events at the end of the movie: 1

Average outcome: 2

Desirable outcome; happy ending; the character achieves his/her goals or experiences happiness and pleasant events at the end of the movie: 3

**Socioeconomic Status* (SES)**:____

Low SES: 1

Average SES: 2

High SES: 3

SES changes from low to high: 4

SES changes from high to low: 5

*The score for pets and toys is based on the SES of the owner. The score for wild animals is unclear; “.”

**Courage**

Character is depicted as cowardly; shows more intense fear and avoidance of unpleasant or dangerous situations than other characters: 1

Character is sometimes courageous and sometimes cowardly: 2

Character is depicted as courageous; is willing to engage in unpleasant or dangerous situations: 3

**Disability** (not including products of magic, like curses)

Cognitive disability that inhibits functioning: 1

Physical disability that inhibits functioning: 2

Neither cognitive nor physical disability that inhibits functioning: 3

**Romantic Involvement**

Attempts to engage in romance and is unsuccessful; rejected: 1

Does not engage in romance: 2

Successfully engages in romance*: 3

Has both successful and unsuccessful romantic encounters: 4

*showing affection or falling in reciprocated love scores a 3; simply being married is not enough to score a 3

**Authority Figure** (A figure of authority is a character who has power over other characters, and who can enforce obedience. Authority figures could include parents, group leaders, politicians, teachers)

The character is in, or was once in, a position of authority: 1

The character has not been in a position of authority: 2

**Motivation**

The character is motivated primarily by self-betterment: 1

The character is motivated both by self-betterment and by the betterment of others: 2

The character is motivated primarily by helping others: 3

**Trustworthiness and Deception**

The character is deceptive; he/she lies or gives an appearance or impression different from the true one; misleading: 1

The character is sometimes trustworthy and sometimes untrustworthy: 2

The character is trustworthy; he/she is able to be relied on as honest or truthful: 3

**Race/ethnicity**

African American/Black: 1

White: 2

Asian/Asian American: 3

Native American/Pacific Islander: 4

Hispanic/Latinx: 5

Other unidentified races/ethnicities: 6

Non-human: 7

Race changes throughout movie: 8
