# Supplementary material for: Moral foundations theory, political identity, and the depiction of morality in children’s movies
Source: PLoS One. 2021 Mar 26;16(3):e0248928. doi: 10.1371/journal.pone.0248928 (PMC7996984; doi:10.1371/journal.pone.0248928)
Supplement: S4 Appendix — (DOCX) [file pone.0248928.s004.docx]

# **S4 Appendix (Moral Dilemma and Resolution Task)**

The following passage is the plot of a children's movie in which a character faces a moral dilemma. One purpose of the movie is to teach children to do the right thing. Please read the passage and answer the following question.

[Text describing the scene/dilemma from each film was presented here]

Imagine it is your job to write the ending of this scene. Below are two hypothetical resolutions to the moral dilemma faced by the character. **Please rate each resolution on a scale from one to seven for the extent to which it would effectively teach children to do the right thing.** A score of seven signifies that the ending is an excellent way to model moral virtues to children. A score of one signifies that the ending is an extremely poor way to model moral virtues to children.

| Extremely poor way to model moral virtues |  |  |  |  |  | Excellent way to model moral virtues |
| --- | --- | --- | --- | --- | --- | --- |
| 1  🔘 | 2  🔘 | 3  🔘 | 4  🔘 | 5  🔘 | 6  🔘 | 7  🔘 |

[Two hypothetical resolutions were presented here]

## **Old Yeller (1957):**

*Travis is teen boy who lives with his family on a farm in the countryside. Travis’s father leaves the farm for three months to sell the family’s steers. Before he leaves, he tells Travis, “Well son, while I’m gone, you’ll be the man of the house.” He describes the obligations that come with providing for a family, including hunting and tending to the farm.*

*One morning some time later, Travis’s mother tells him to shoot a deer for dinner. He waits in some cover near a stream, and hears a noise. He aims his rifle in the direction of the noise, and sees two deer emerge from the bushes. It appears to be a mother and fawn, and they drink from the stream without noticing him.*

• Travis shoots the deer because his mother told him to, and to provide for his family (the scene would not depict the deer being shot but instead imply that Travis pulled the trigger). [Binding-promoting resolution]

• Travis does not shoot the deer because it has a fawn, and it is right to show compassion to animals. [Individualizing-promoting resolution]

## **Short Circuit (1986):**

*Nova Robotics is a company that designs human-like robots for combat use in the military. One day, one of the robots is struck by lightning and malfunctions, becoming able to move and behave independently of humans. It leaves the company grounds and wanders through the city. It is eventually taken in by Stephanie, a baker who lives in the area. Stephanie gives the robot, which she calls Number 5, a dictionary. Number 5 uses the dictionary to learn English, and also appears to enjoy watching TV and dancing.*

*Stephanie soon realizes that the robot belongs to Nova Robotics, and she calls the company requesting that they come pick it up. The company managers are eager to fix the malfunction so that Number 5 can serve its original purpose. When Stephanie explains to Number 5 that it will be taken back to Nova Robotics for a “tune up,” it becomes visibly agitated, saying “No disassemble!” and “Number 5 is alive!”. Number 5 tries to escape.*

• Stephanie helps return Number 5 to the company, so it can be used to benefit the military. [Binding-promoting resolution]

• Stephanie helps Number 5 escape, because it indicates that it does not want to be harmed. [Individualizing-promoting resolution]

## **Moana (2016):**

*Chief Tui and his family live in a small village on an island. The villagers have a rich culture and traditions that are an integral part of life. One such tradition is that fishermen always fish in a few designated areas. One day, Chief Tui’s daughter Maya proposes a new fishing location. Chief Tui explains that the location she suggested is a particularly dangerous area, and forbids her from trying to fish there.*

*Maya believes that the new fishing location she proposed is better than the traditional areas. Contrary to her father's wishes, she takes a small fishing vessel to try to reach this alternative fishing location. The waves are rough. She loses control of the boat, falls into the water, and has to swim to shore. When she and the vessel wash back to shore, Maya finds that her Grandma has witnessed what happened from the beach. Worried about being punished, Maya requests that her Grandma not tell Chief Tui what happened.*

• The Grandmother tells Maya’s father what happened to ensure that, in the future, Maya listens to her father and the village’s traditions are preserved. [Binding-promoting resolution]

• The Grandmother does not tell Maya’s father because Maya has the right to explore, and should be protected from punishment. [Individualizing-promoting resolution]

## **Treasure Island (1950):**

*Jim is a boy who lives on a ship with a crew of sailors. The Captain of the ship is a respected man on a journey to find buried treasure by following a treasure map to an island. The Captain knows that the treasure was buried there by pirates, and wants to claim it before anyone else does. The Captain is fond of Jim and counts on him to be a loyal contributor of the crew. The Captain and the crew are like a family to Jim.*

*One of Jim’s friends on the ship is Sam. Sam is an impoverished sailor with a peg leg who has shown kindness to Jim in the past. One day, Jim overhears Sam planning with another sailor to steal the treasure map from the Captain and embark on a mission to find the treasure without the rest of the crew. Jim has to decide whether to tell the Captain of Sam’s betrayal.*

• Jim tells the Captain what Sam is planning, because it is his duty to be loyal to the Captain and the crew. [Binding-promoting resolution]

• Jim does not tell the Captain what Sam is planning, because it might result in harm coming to Sam, and it is unfair for the Captain to get the treasure when he is already rich. [Individualizing-promoting resolution]
