# Supplementary material for: Moral foundations theory, political identity, and the depiction of morality in children’s movies
Source: PLoS One. 2021 Mar 26;16(3):e0248928. doi: 10.1371/journal.pone.0248928 (PMC7996984; doi:10.1371/journal.pone.0248928)
Supplement: S5 Appendix — (DOCX) [file pone.0248928.s005.docx]

# **S5 Appendix (Analyses Reported in Study 1)**

**Individualizing foundations among heroes vs. villains**

summary(lm(indiv_avg~Villain,data=Study1_data))

**
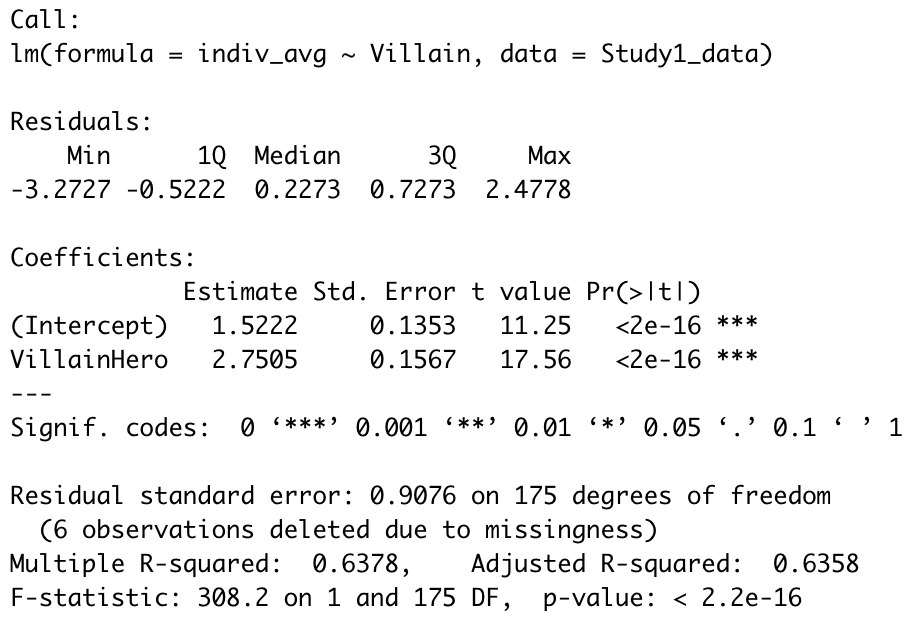
**

**Binding foundations among heroes vs. villains**

summary(lm(binding_avg~Villain,data=Study1_data))


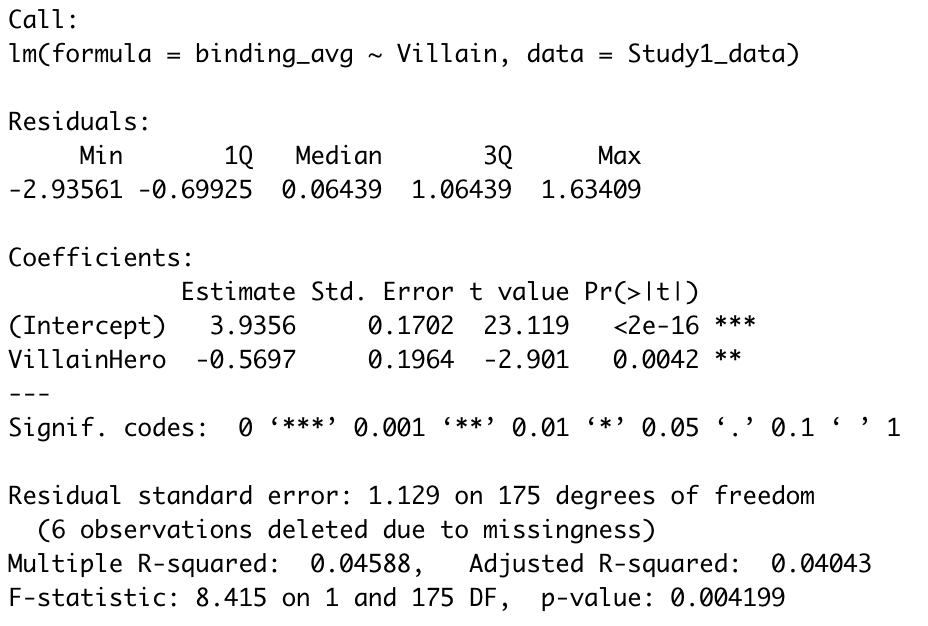


**Individualizing vs. binding foundations within heroes**

summary(lm((indiv_avg-binding_avg)~1,data=Study1_data,subset=Villain=="Hero"))

**
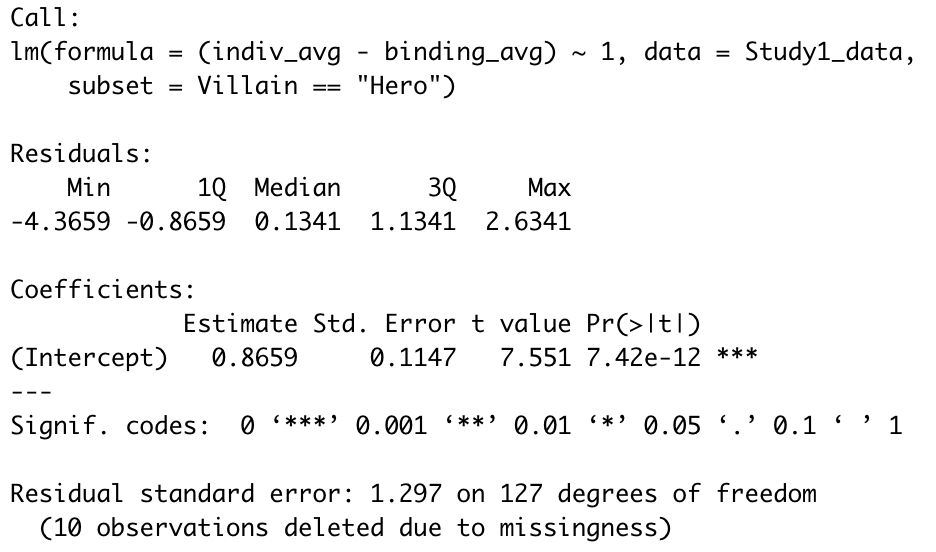
**

**Individualizing vs. binding foundations within villains**

summary(lm((indiv_avg-binding_avg)~1,data=Study1_data,subset=Villain=="Villain"))

**
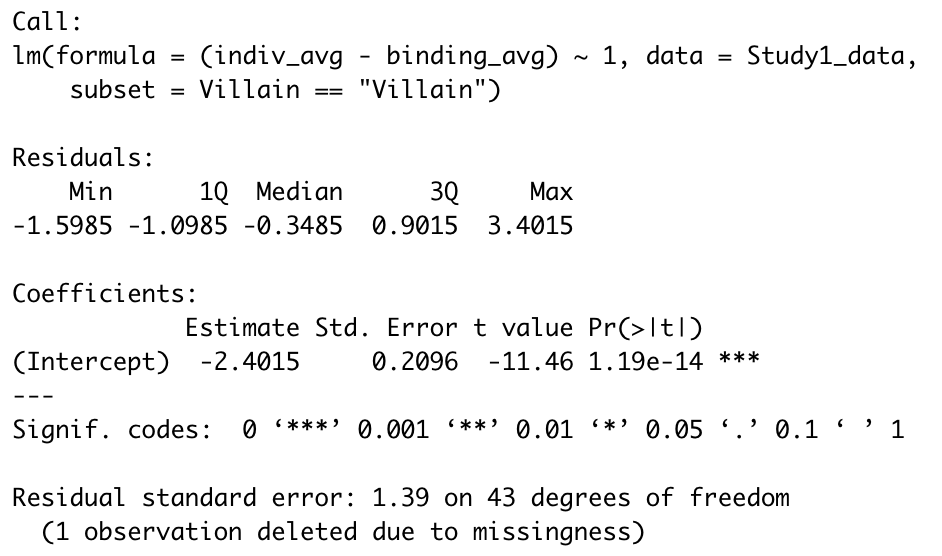
**

**Hero vs. villain comparison for each of the five MFT foundations**

summary(lm(Care~Villain,data=Study1_data))


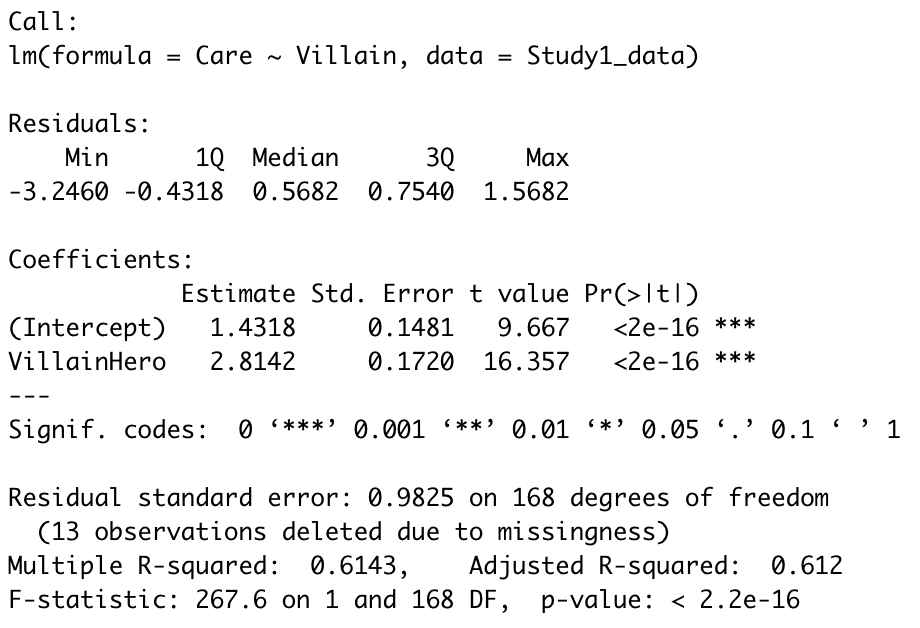


summary(lm(Fairness~Villain,data=Study1_data))


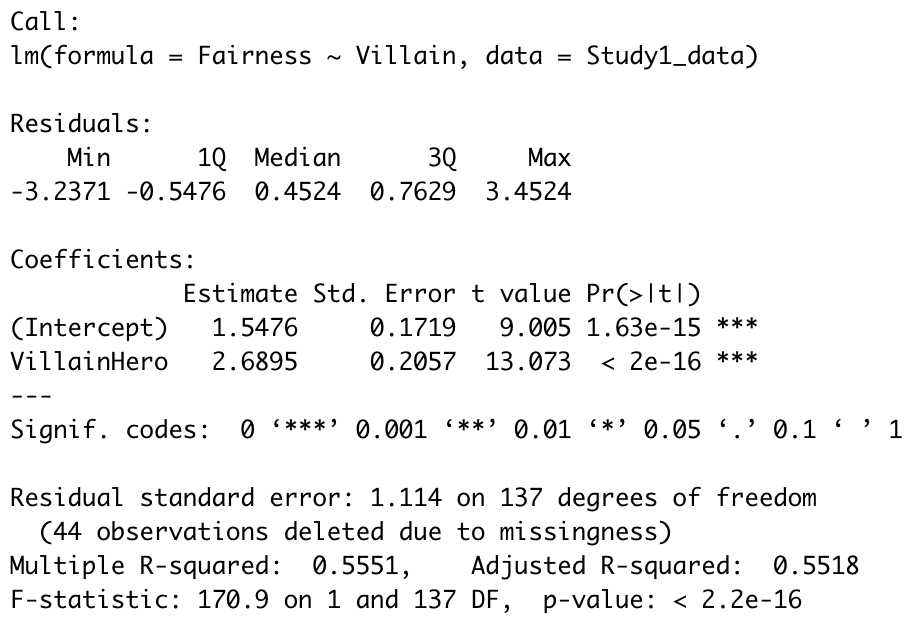


summary(lm(Authority~Villain,data=Study1_data))


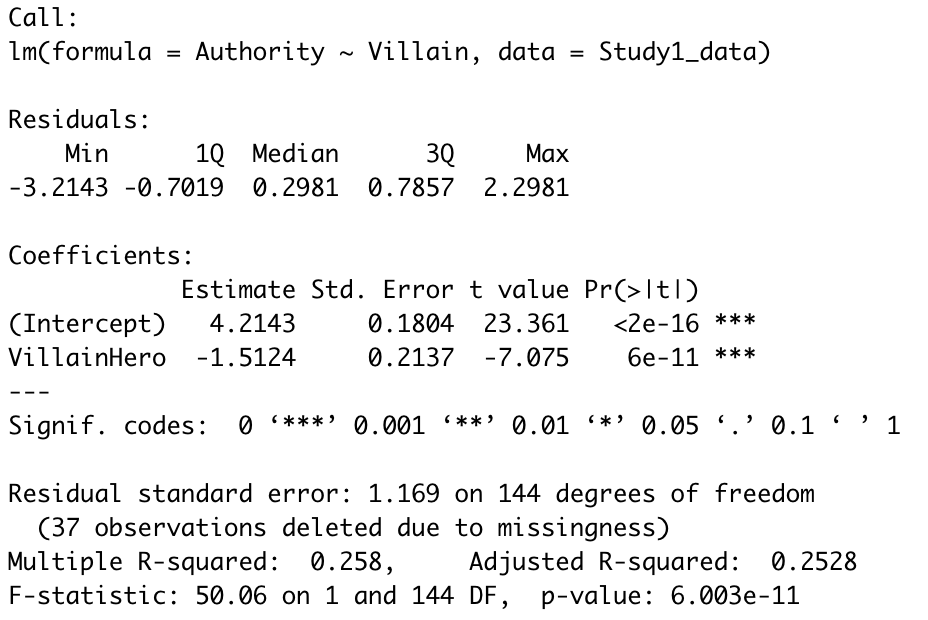


summary(lm(Loyalty~Villain,data=Study1_data))


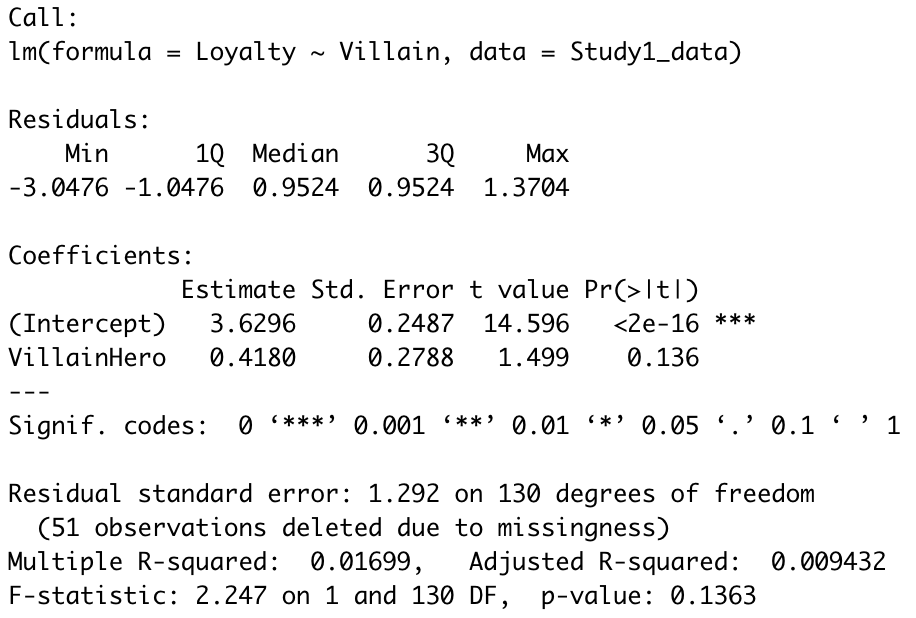


summary(lm(Sanctity~Villain,data=Study1_data))


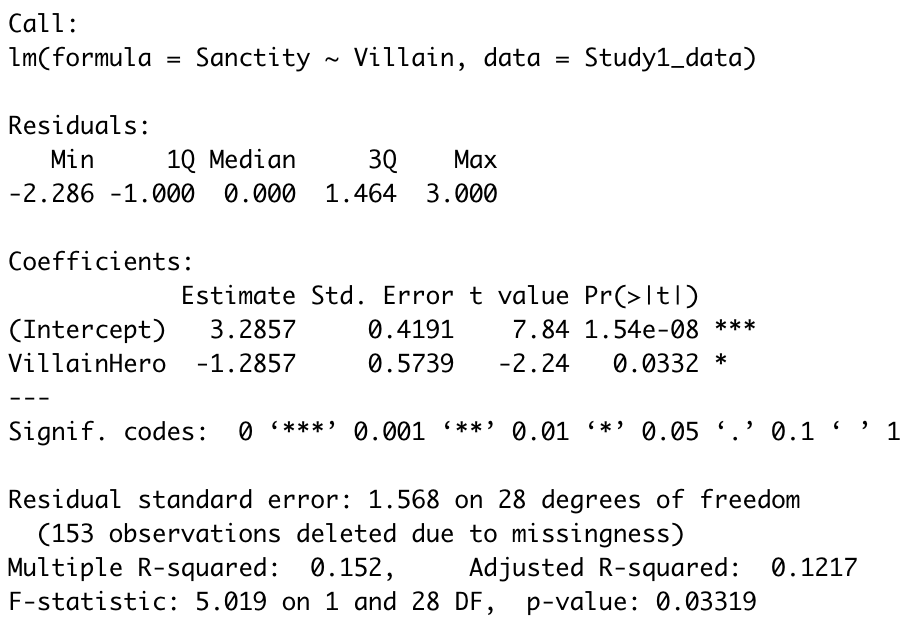


**Additional characteristics as predictors of individualizing foundations**

indiv_outcome<-t.test(indiv_avg~Outcome,data=Study1_data,subset=Outcome=="Undesirable"|Outcome=="Desirable")

indiv_attract<-t.test(indiv_avg~Attractiveness,data=Study1_data,subset=Attractiveness=="Attractive"|Attractiveness=="Unattractive")

indiv_ses<-t.test(indiv_avg~SES,data=Study1_data,subset=SES=="Low"|SES=="High")

indiv_violence<-t.test(indiv_avg~Violence,data=Study1_data,subset=Violence=="Violent"|Violence=="Nonviolent")

indiv_accent<-t.test(indiv_avg~Accent,data=Study1_data,subset=Accent=="Accent"|Accent=="No_Accent")

indiv_authfig<-t.test(indiv_avg~Authority_Figure,data=Study1_data,subset=Authority_Figure=="Authority"|Authority_Figure=="Non-Authority")

indiv_gender<-t.test(indiv_avg~Gender,data=Study1_data,subset=Gender=="Male"|Gender=="Female")

indiv_romantic<-t.test(indiv_avg~Romantic_Involvement,data=Study1_data,subset=Romantic_Involvement=="Successful"|Romantic_Involvement=="Unsuccessful")

indiv_weight<-t.test(indiv_avg~Weight,data=Study1_data,subset=Weight=="Overweight/Obese"|Weight=="Average Weight")

indiv_race<-t.test(indiv_avg~Race,data=Study1_data,subset=Race=="White"|Race=="Non-human")

**Bejamini-Hochberg corrected *p*-values from the 10 analyses above**

p.adjust(c(indiv_outcome$p.value,indiv_attract$p.value,indiv_ses$p.value,indiv_violence$p.value,indiv_accent$p.value,indiv_authfig$p.value,indiv_gender$p.value,indiv_romantic$p.value,indiv_weight$p.value,indiv_race$p.value),method="BH")

**Additional characteristics as predictors of binding foundations**

bind_outcome<-t.test(binding_avg~Outcome,data=Study1_data,subset=Outcome=="Undesirable"|Outcome=="Desirable")

bind_attract<-t.test(binding_avg~Attractiveness,data=Study1_data,subset=Attractiveness=="Attractive"|Attractiveness=="Unattractive")

bind_ses<-t.test(binding_avg~SES,data=Study1_data,subset=SES=="Low"|SES=="High")

bind_violence<-t.test(binding_avg~Violence,data=Study1_data,subset=Violence=="Violent"|Violence=="Nonviolent")

bind_accent<-t.test(binding_avg~Accent,data=Study1_data,subset=Accent=="Accent"|Accent=="No_Accent")

bind_authfig<-t.test(binding_avg~Authority_Figure,data=Study1_data,subset=Authority_Figure=="Authority"|Authority_Figure=="Non-Authority")

bind_gender<-t.test(binding_avg~Gender,data=Study1_data,subset=Gender=="Male"|Gender=="Female")

bind_romantic<-t.test(binding_avg~Romantic_Involvement,data=Study1_data,subset=Romantic_Involvement=="Successful"|Romantic_Involvement=="Unsuccessful")

bind_weight<-t.test(binding_avg~Weight,data=Study1_data,subset=Weight=="Overweight/Obese"|Weight=="Average Weight")

bind_race<-t.test(binding_avg~Race,data=Study1_data,subset=Race=="White"|Race=="Non-human")

**Bejamini-Hochberg corrected *p*-values from the 10 analyses above**

p.adjust(c(bind_outcome$p.value,bind_attract$p.value,bind_ses$p.value,bind_violence$p.value,bind_accent$p.value,bind_authfig$p.value,bind_gender$p.value,bind_romantic$p.value,bind_weight$p.value,bind_race$p.value),method="BH")
