# Supplementary material for: Moral foundations theory, political identity, and the depiction of morality in children’s movies
Source: PLoS One. 2021 Mar 26;16(3):e0248928. doi: 10.1371/journal.pone.0248928 (PMC7996984; doi:10.1371/journal.pone.0248928)

# **S6 Appendix (Analyses Reported in Study 2)**

**Political orientation as predictor of individualizing-MFQ scores**

summary(lm(Study2_data$MFQ_Indiv~Study2_data$Political_LibLeft))


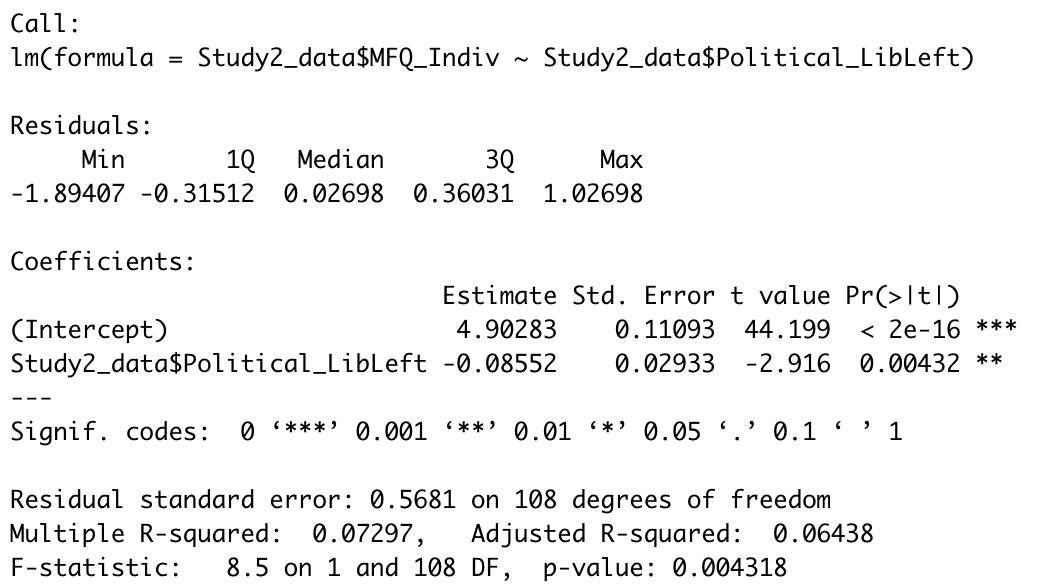


**Political orientation as predictor of binding-MFQ scores**

summary(lm(Study2_data$MFQ_Binding~Study2_data$Political_LibLeft))


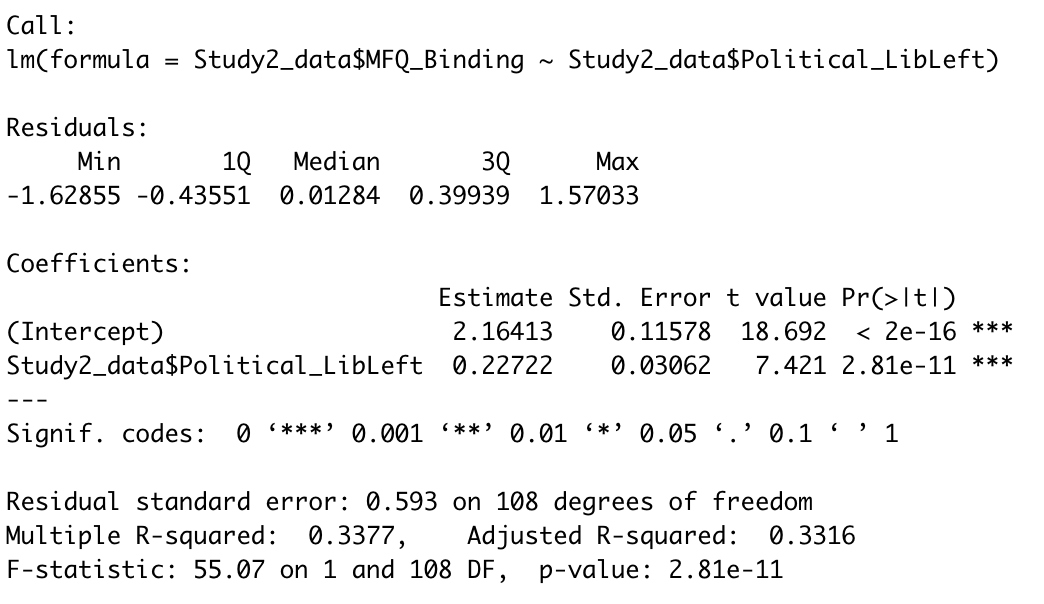


**Intercorrelations among MFQ scores and political orientation**

iMFQ_pol_r<-abs(cor(Study2_data$MFQ_Indiv,Study2_data$Political_LibLeft))

bMFQ_pol_r<-abs(cor(Study2_data$MFQ_Binding,Study2_data$Political_LibLeft))

iMFQ_bMFQ_r<-abs(cor(Study2_data$MFQ_Indiv,Study2_data$MFQ_Binding))

**Comparing the difference in magnitude between the individualizing-MFQ and politics correlation vs. the binding-MFQ and politics correlation**

library(cocor)

cocor.dep.groups.overlap(iMFQ_pol_r,bMFQ_pol_r,iMFQ_bMFQ_r,n=110)


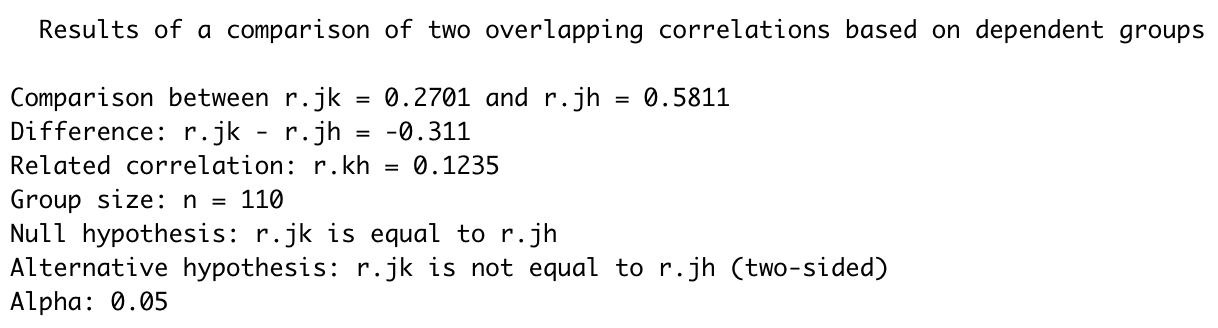


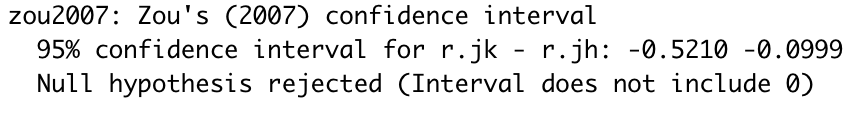


**Intercorrelations among MFQ scores and resolution scores**

cor.test(Study2_data$Resol_Indiv,Study2_data$MFQ_Indiv)


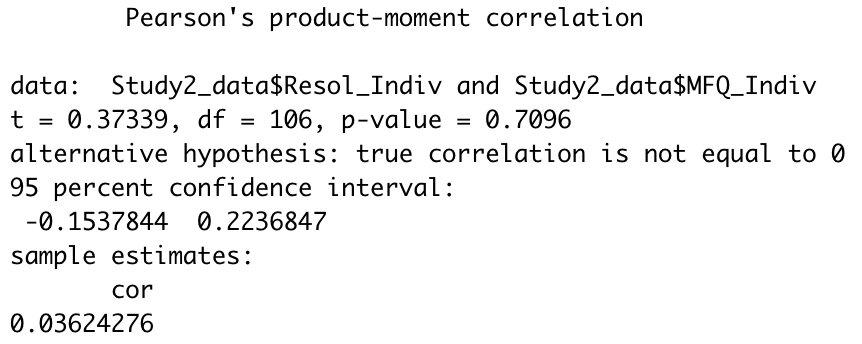


cor.test(Study2_data$Resol_Indiv,Study2_data$MFQ_Binding)


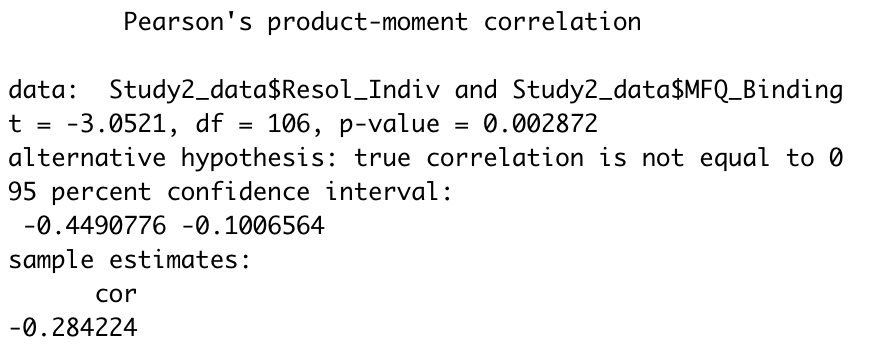


cor.test(Study2_data$Resol_Binding,Study2_data$MFQ_Indiv)


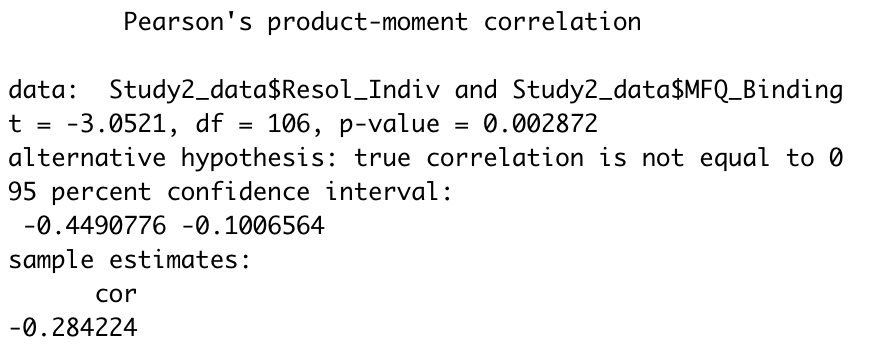


cor.test(Study2_data$Resol_Binding,Study2_data$MFQ_Binding)


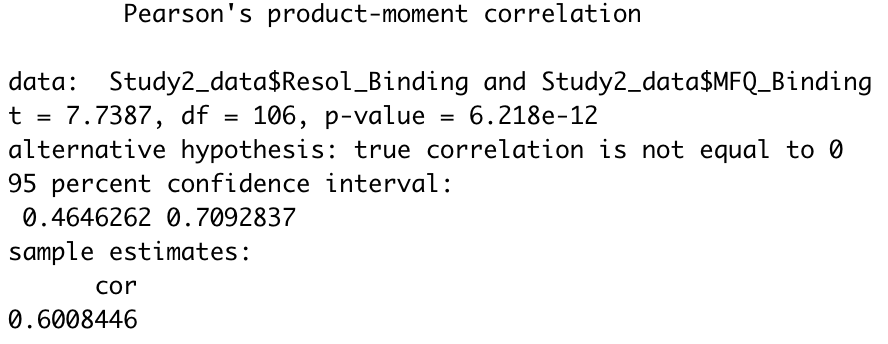


**Political orientation as predictor of individualizing-resolution scores**

summary(lm(Study2_data$Resol_Indiv~Study2_data$Political_LibLeft))


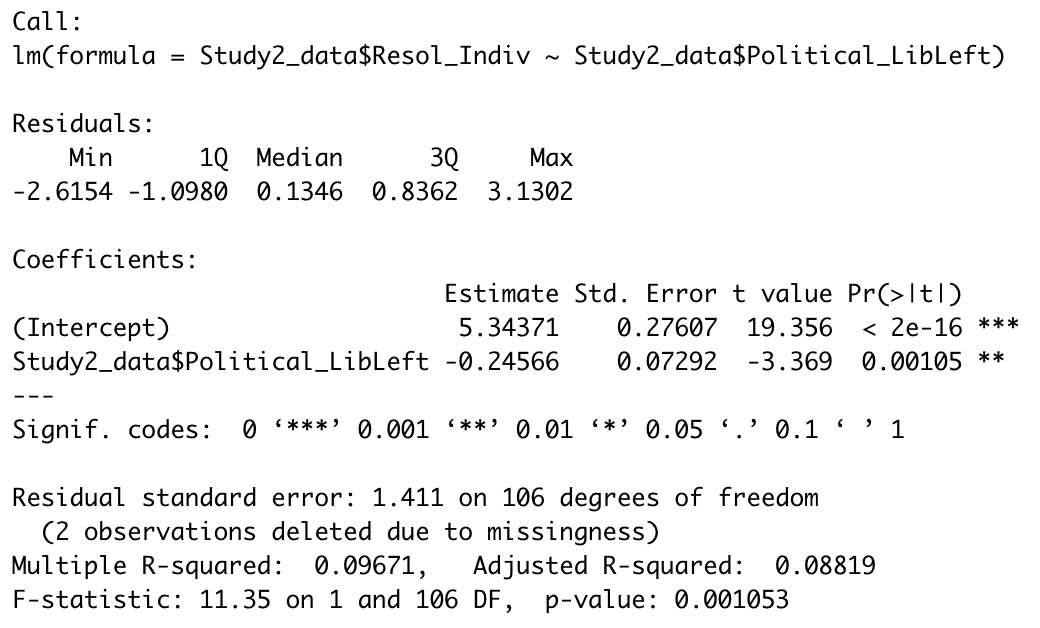


**Political orientation as predictor of binding-resolution scores**

summary(lm(Study2_data$Resol_Binding~Study2_data$Political_LibLeft))


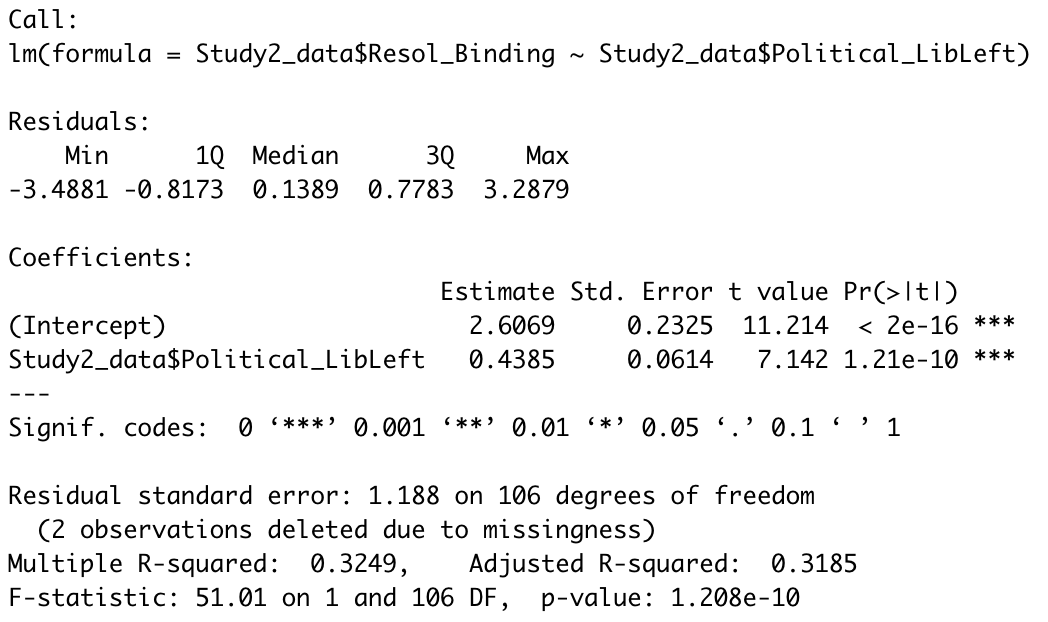


**Intercorrelations among resolution scores and political orientation**

iResol_pol_r<-abs(cor(Study2_data$Resol_Indiv,Study2_data$Political_LibLeft,use="p"))

bResol_pol_r<-abs(cor(Study2_data$Resol_Binding,Study2_data$Political_LibLeft,use="p"))

iResol_bResol_r<-abs(cor(Study2_data$Resol_Indiv,Study2_data$Resol_Binding,use="p"))

**Comparing the difference in magnitude between the individualizing-resolution and politics correlation vs. the binding-resolution and politics correlation**

library(cocor)

cocor.dep.groups.overlap(iResol_pol_r,bResol_pol_r,iResol_bResol_r,n=108)

**
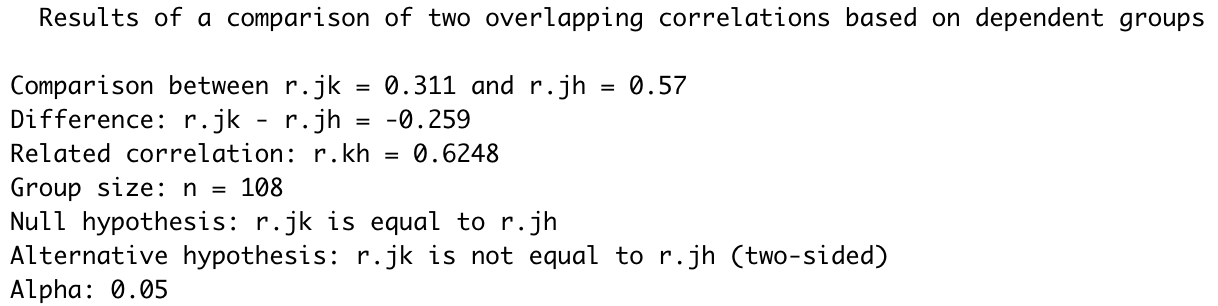
**

**
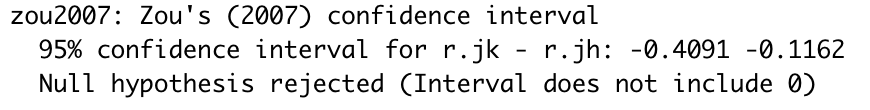
**

**Regressions predicting resolution scores by political orientation, controlling for MFQ scores**

summary(lm(Resol_Indiv~Political_LibLeft+MFQ_Indiv+MFQ_Binding,data=Study2_data))


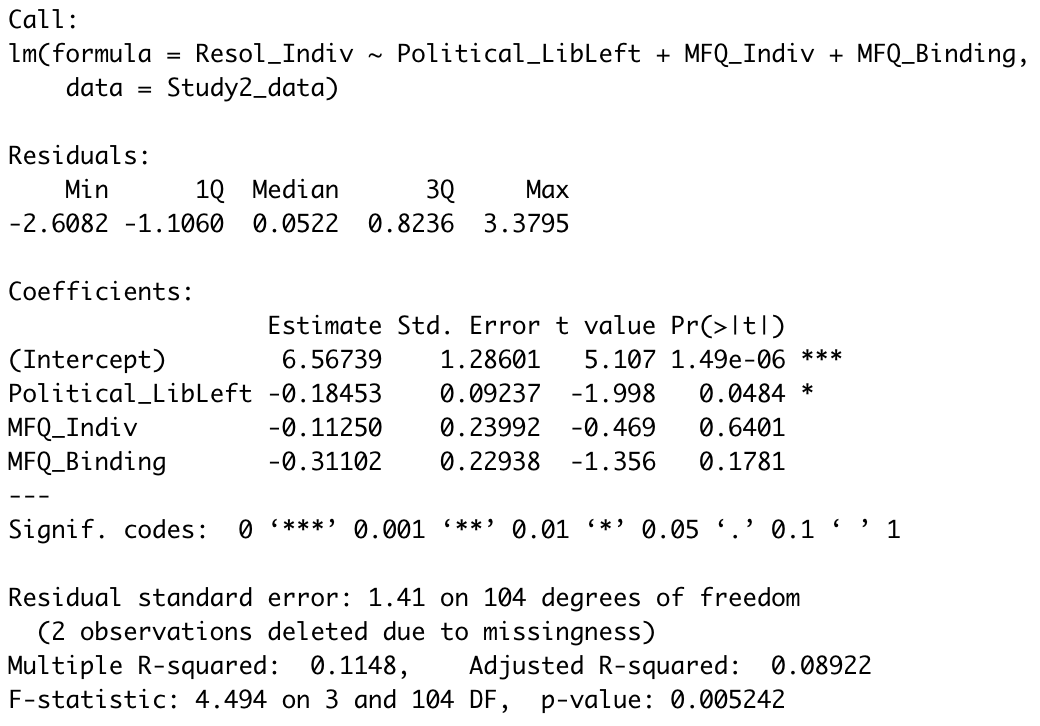


summary(lm(Resol_Binding~Political_LibLeft+MFQ_Indiv+MFQ_Binding,data=Study2_data))


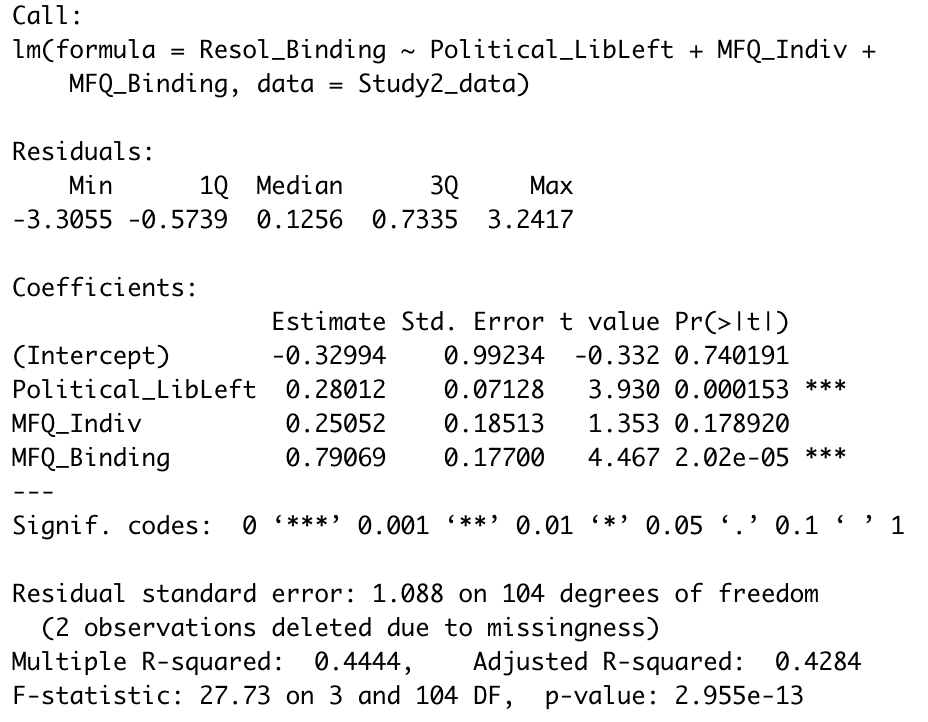

Supplement: S6 Appendix — (DOCX) [file pone.0248928.s006.docx]
