# Supplementary material for: Moral foundations theory, political identity, and the depiction of morality in children’s movies
Source: PLoS One. 2021 Mar 26;16(3):e0248928. doi: 10.1371/journal.pone.0248928 (PMC7996984; doi:10.1371/journal.pone.0248928)

# **S7 Appendix (Supplementary Analyses for Study 2)**

**Mixed models examining political orientation x foundation category interaction**

The *cocor* analyses in Study 2 showed that the correlation between political orientation and individualizing concerns was of weaker magnitude than the correlation between political orientation and binding concerns (this was true for both MFQ scores and resolution scores). We also ran a conceptually similar analysis: a mixed-model regression with an interaction term. To do so, we transformed the original dataset into a new one in which each subject occupied two rows (one for each umbrella category of MFT domains: individualizing and binding), with columns to represent their MFQ endorsement scores and their resolution endorsement scores, as well as a column representing their (standardized) political orientation. Notably, if we ran the regression model at this point, it would undoubtedly reveal a political orientation x foundation category interaction, but this would merely reflect the fact that the correlations between political orientation and the MFT categories have different signs (stronger conservatism associated with less endorsement of individualizing foundations and more endorsement of binding foundations).

To be able to assess whether the magnitudes of the correlations differed instead , we inverted participants’ individualizing ratings (computing these new inverted scores as 7 minus their original score in the case of MFQ endorsement, and 8 minus their original score in the case of resolution endorsement, since the original scores were assessed on a 1 to 6 scale and a 1 to 7 scale, respectively). Thus, higher scores on these new inverted scores represent *less* endorsement of the individualizing foundations. Stronger conservatism is therefore positively correlated with both binding scores and with inverted individualizing scores; the interaction term in the model now assesses whether these two correlations differ in magnitude.

We ran two separate regression models — one for MFQ scores and one for resolution scores — predicting endorsement as a function of (standardized) political orientation, foundation category, and their interaction (again, such that individualizing ratings were inverted), including random intercepts for subjects. Both analyses revealed a significant effect of political orientation (stronger conservatism predicted stronger endorsement of binding foundations and of inverted individualizing foundations) and of foundation type (this effect is not conceptually very meaningful because it compares whether endorsement of binding foundations differs from endorsement of inverted individualizing foundations).

Both analyses revealed a significant political orientation x foundation category interaction, revealing that the correlation between political orientation and binding endorsement was stronger than that between political orientation and (inverted) individualizing endorsement. In other words, as compared to endorsement of binding foundations, there is relatively more agreement in the importance of individualizing foundations across political orientation. These regression analyses therefore lead to the same conclusions as those from the *cocor* analyses.

library(lme4)

summary(lmer(resol_rating~zPolitical_LibLeft*foundation_num+(1|subj),data=Study2_data_hlm))


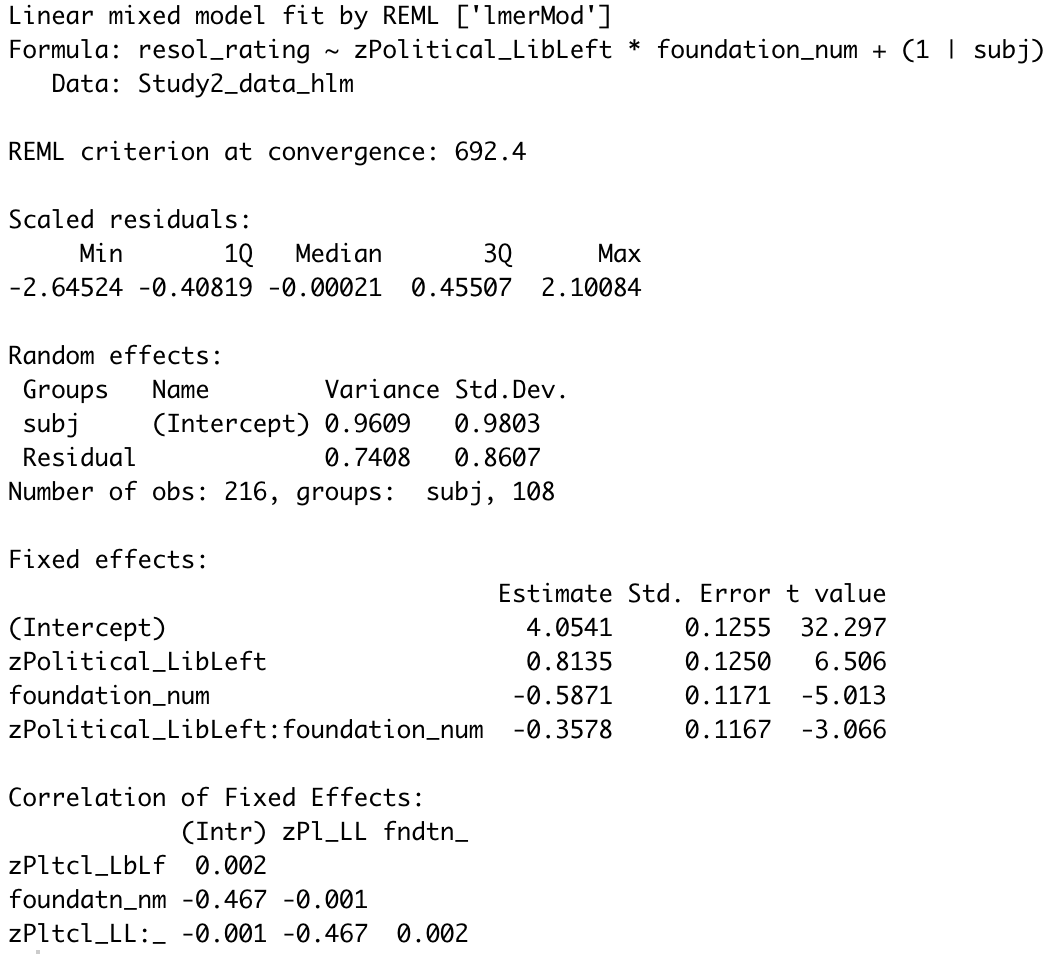


summary(lmer(MFQ_rating~zPolitical_LibLeft*foundation_num+(1|subj),data=Study2_data_hlm))


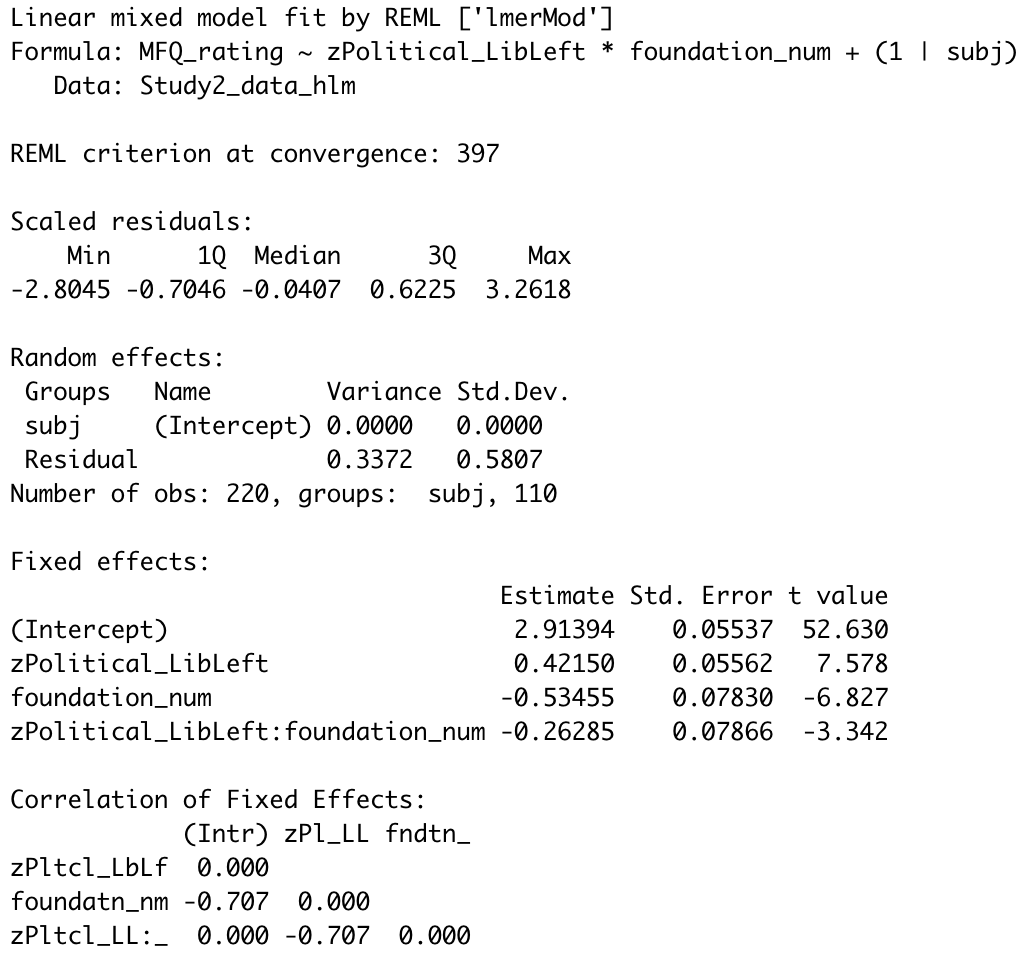

Supplement: S7 Appendix — (DOCX) [file pone.0248928.s007.docx]
