# Supplementary material for: Moral foundations theory, political identity, and the depiction of morality in children’s movies
Source: PLoS One. 2021 Mar 26;16(3):e0248928. doi: 10.1371/journal.pone.0248928 (PMC7996984; doi:10.1371/journal.pone.0248928)
Supplement: Attachment — Submitted filename: Comments from reviewer.docx [file pone.0248928.s008.docx]

The paper reports two studies related to the moral foundations theory. The first one found that heroes in children’s movie characters emphasize individualizing foundations whereas villains demonstrate binding foundations. The second study links preference for a certain moral foundation to the political self-identification. Although the two studies might be interesting both have serious limitations, and the paper as a whole is perhaps not ready for publication.

Major concerns:

1. The two studies only vaguely related. They don’t address a single problem; they answer very different questions. In particular, the paper doesn’t contain a common aim or a general problem. The paper doesn’t even have an Introduction section that could explain the connection between the two studies. It seems weird to begin the paper with the literature review without knowing the problem under consideration. Due to the lack of common ground for the two studies the paper can be confusing. For example, the second sentence of the abstract points to political differences - unclear why. The added value of the paper is questionable as well, since it’s not clear what literature it is supposed to contribute to: developmental psychology? political science? media studies? As long as the field, and, more importantly, a single research question is defined, the literature review and a (future) introduction should clearly state what particular problem is being solved by the current paper.

2. Each study taken separately is underdeveloped (see below). If the authors consider further development of the paper, I’d suggest separating it into two different papers and significantly expanding each study. For instance, both studies would benefit from including them into a rich context of communication research, which addressed similar questions for decades, to mention a few: Grizzard, M., Huang, J., Fitzgerald, K., Ahn, C., & Chu, H. (2018). Sensing heroes and villains: Character-schema and the disposition formation process. Communication research, 45(4), 479-501; Grizzard, M., Fitzgerald, K., Francemone, C. J., Ahn, C., Huang, J., Walton, J., ... & Eden, A. (2020). Validating the extended character morality questionnaire. Media Psychology, 23(1), 107-130. Matthews, N. L. (2019). Detecting the boundaries of disposition bias on moral judgments of media characters’ behaviors using social judgment theory. Journal of Communication, 69(4), 418-441. Hastings, A. W. (1993). Moral Simplification in Disney's The Little Mermaid. The Lion and the Unicorn, 17(1), 83-92. Padilla-Walker, L. M., Coyne, S. M., Fraser, A. M., & Stockdale, L. A. (2013). Is Disney the nicest place on earth? A content analysis of prosocial behavior in animated Disney films. Journal of Communication, 63(2), 393-412.

3. Both studies are presented (until conclusion) in a U.S.-centered manner, including movies, participants, political culture etc. It’s worth mentioning the kind of participants in Study 2 (U.S. adults) and the sample of movies (American) in Study 1. The fact that movies studied were all U.S.-produced needs emphasis, as in other cultures the results would likely differ (e.g. consider Indian cinematography where villains are likely to be individualists, whereas heroes might stick to bonding foundations).   The related issue is a representativeness. Both studies use samples and it’s unclear what general population (of movies or persons) they are supposed to represent.   In particular, in Study 1:

1. How movies were identified as ‘children’s’ rather than ‘general population’s’?

2. Why the first year of selection was specifically 1948?

3. Why top grossing movies were selected rather than e.g. top-rated? What the grossing is supposed to represent?

4. Why movies and not characters were sampled?

5. Why only (and exactly) seven movies were selected in the end? Are they somehow representative of all the movies.

These are important questions as the authors generalize, e.g. ‘These findings reveal that the moral messages conveyed in children’s media…’ should then read ‘These findings reveal that the moral messages conveyed in SEVEN AMERICAN MOVIES…’ and it changes the meaning of conclusions dramatically.

In Study 2, the participants come from a convenience sample, therefore all the conclusions should be bound to these specific people rather than to any other population. The results should not be generalized to ‘people in general’ (p.21) or ‘liberals’ or ‘conservatives’ (p.22) in general, because the sample is not representative of any of these groups. The conclusion connects these results to parenting, but the sample doesn’t seem to represent parents as well. What was a median age of the participants?

Minor issues:

* Throughout the paper the moral foundations are equated with moral values (and ‘relevance’ with ‘importance’), which are not exactly synonyms. ‘Scores of 5 indicated a character clearly and consistently valued the given foundation’, whereas MFQ (Graham et al, 2011) includes ‘relevance’ and ‘agreement’, not importance . THis is one issue that makes a difference between foundations which a composite construct (syndrome of social norms, attitudes, beliefs, of which values are only a small part) and values which is much more specific construct (see e.g. Schwartz, S. H. (1992). Universals in the content and structure of values: Theoretical advances and empirical tests in 20 countries. Advances in experimental social psychology, 25(1), 1-65.)

* Footnote 2: I’d disagree that coders’ guessing what ‘the film clearly intended’ is able to remove personal bias. What would, indeed, remove personal bias is an explicit characteristics given to a character unequivocally by the other characters of the film. Overall, authors’ intention is an outdated construct disproved as early as in the 1960s (e.g. by Roland Barthe).

* Given there were only two raters, it would be beneficial to mention their gender, race, and nationality. Is there any evidence that children’s perceptions are compatible with those made by the two raters?

* The coding scheme is ambiguous sometimes. For example, Care/Harm includes ‘Is sensitive to the emotions of others’ vs ‘Indifference to the suffering’ whereas many villains are sadistic, that is, they’re not indifferent rather they enjoy suffering of others.

* The data provided do not contain the raw scores given by each each rater, rather it seems to contain single rater’s responses. It makes, e.g. inter-rater reliability test non-replicable. The ‘movie’ variable is empty.

* One issue in Study 2 can be related to the use of existing movie stories as stimuli. The familiarity with the movie or a character in general likely affected responses.

* What makes a difference between heroes and villains in general? Perhaps their morals is what defines this distinction and not the other way around. THat is, it might be that it’s not binding foundation is presented negatively, but it is the very thing that defines a villain.
