# Supplementary material for: Moral foundations theory, political identity, and the depiction of morality in children’s movies
Source: PLoS One. 2021 Mar 26;16(3):e0248928. doi: 10.1371/journal.pone.0248928 (PMC7996984; doi:10.1371/journal.pone.0248928)
Supplement: Attachment — Submitted filename: PLoS ONE Revision response to reviewers.docx [file pone.0248928.s009.docx]

Professor Valerio Capraro

Academic Editor, *PLoS ONE*

February 24, 2021

Dear Professor Capraro:

Thanks to you and the reviewers for consideration of our manuscript PONE-D-20-26290, “Moral Foundations Theory, Political Ideology, and the Depiction of Moral Values in Children’s Movies” (note that in our enclosed revision, we have slightly revised this original title). We appreciate the time and effort you and the reviewers put into our work, and we are grateful for your thoughtful feedback. We have made careful and extensive revisions in response to the reviewers’ excellent comments, and we believe our manuscript is greatly improved as a result.

We detail the specifics of our revisions beginning on the next page, with reviewer comments *in italics* followed by our response in plain text.

As you requested, we are enclosing a marked-up copy of the manuscript that highlights changes made to the original (labeled ‘Revised Manuscript with Track Changes’), as well as an unmarked version without track changes (labeled ‘Manuscript’).

My co-authors and I look forward to hearing from you.

Sincerely,

Steve Guglielmo

Associate Professor of Psychology

In this document, we detail the specifics of our revisions, with reviewer comments *in italics* followed by our response in plain text.

**Reviewer 1**

*1. In Study 1, the authors first choose the most popular movies per decade and then sample only one per decade to analyze (7 movies in total have been analyzed). I am not an expert in the content analysis of this kind but a rather small sample of movies being analyzed made me wonder of how “representative” it might be for children’s movies in US/North America as such. Especially given the fact that the movies were selected from such a broad period of time. Is it a standard procedure to select only a few movies for the analysis?*

*Although the analysis of changes in the characters and the moral codes they have been conveying could be an interesting follow up, I was wondering if it would not serve the purpose of the study better if the authors focus on the analysis of only the recent movies?*

We now realize that we were not clear enough in the original manuscript. Our complete dataset of characters/movies contained 183 characters from 42 movies. All of our hypothesis testing (and exploratory analyses) were conducted on this complete dataset.

To ensure adequate consistency in the use of the coding scheme, we examined inter-rater reliability using a subset of characters/movies (41 characters from seven randomly selected movies). The only analyses performed on this subset where those used to assess inter-rater reliability (Kappa and ICC). We apologize for the lack of clarity surrounding this method in our initial submission and have worked to clarify it in the revised manuscript.

*2. I have also concerns related to the sample in Study 2:*

*2a. In my opinions, important details are missing with regard to who has been invited to take part in Study 2 and the final sample (what is the age, education, and nationality of the participants?).*

*2b. How the sample size was determined? E.g., what was the stopping rule for the data collection?*

*These two issues make me hesitant about whether we can draw strong conclusions from this research.*

Our stopping rule for data collection was based on time: we planned to obtain as many participants as possible over an approximately two-week period. Given the reviewer’s comment, we conducted a post hoc power analysis to determine our ability to detect a significant effect, given our sample of *N*=110. Graham et al. (2011) reported that, among US participants, the correlation between political ideology and moral foundation endorsement was at least *r*=.35 (for the harm foundation; all other foundations had correlations of higher magnitude). Our post hoc power analysis suggests we had 96% power to detect a correlation of *r*=.35 with a sample of *N*=110.

We did not collect participants’ education or nationality in this study. Examination of the IP addresses, which are automatically recorded by Qualtrics, revealed that 98% of the sample (108 of 110 participants) completed the study from an IP address within the United States.

The average age of participants in this study was 32.14 years old (*SD* = 15.68).

The composition of our sample is fairly consistent with the composition of online samples, including those recruited via MTurk (Buhrmester, Kwan, & Gosling, 2011; Paolacci & Chandler, 2014).

*3. Although the study is ran in the US context, this issue has not been raised much (e.g., as mentioned earlier, the nationality of the sample is not stated in Study 2). Although the authors suggest that the findings might be different in the collectivistic cultures, in my opinion this discussion was framed in a way that the “default” option of how things work is presented in the current paper and other cultures or contexts are some sort of exceptions (see for example, Cheon, Melani, & Hong, 2020). I find this problematic and would like to encourage authors to think about what are the boundaries to the generalizability of their findings.*

*Cheon, B. K., Melani, I., & Hong, Y. Y. (2020). How USA-Centric Is Psychology? An Archival Study of Implicit Assumptions of Generalizability of Findings to Human Nature Based on Origins of Study Samples. Social Psychological and Personality Science, 11(7), 928-937.*

Thank you for raising this very important issue. We now clarify in several places (including the Introduction and General Discussion) that we are examining morality specifically in a US context. We have also added a paragraph to the General Discussion in which we discuss possible cultural differences in the patterns we have examined. In particular, we think there is good reason to expect both that endorsement of moral values differ across cultural contexts (such that heroic characters in collectivistic cultures will value the binding foundations more than those in American movies), and also that political orientation is robustly and systematically associated with moral foundations across cultures.

*4. The hypotheses to Study 1 are not clear to me: on p. 7 and also on p. 8, they refer to the differences between heroes and villains (e.g., p.7: “heroes would value individualizing foundations more strongly than villains, whereas the opposite would be true for binding foundation”), whereas later in the Results section, the authors present between-characters differences as well as within-characters differences for the two kinds of moral foundations. Please clarify what kind of differences (between- or within-protagonists) you were expecting and why.*

Our hypotheses indeed pertain to the differences between heroes and villains. Therefore, the between-character comparisons (heroes vs. villains) are our primary focus. We think that there nonetheless is value in presenting the within-character comparisons, so we still include these, although we now clarify that they were secondary questions of interest that do not directly pertain to our primary hypotheses.

*5a. Furthermore, when the foundations are analyzed separately, the authors focus only on the differences between heroes and villains but not differences within the two types of characters.*

Consistent with the typical approach in the literature, we believe that the most appropriate way to examine between-foundation differences (and thus within-character patterns) is to compare the set of individualizing foundations to the set of binding foundations. We don’t think it would be especially meaningful (and it certainly wouldn’t be typical) to compare each separate foundation to each other. Therefore we report the individualizing vs. binding comparison for both heroes and villains, but we are reluctant to conduct and report analyses comparing each separate foundation to each other.

*5b. Relatedly, the analysis for the Study 1 could be, in my opinion, more elegant if instead of running three t-tests (p. 11), the authors run on ANOVA with an interaction.*

We appreciate this suggestion, but in this case we think the pairwise comparisons are important (i.e., comparing the hero vs. villain difference for both individualizing and binding foundations, as well as the individualizing vs. binding difference for both heroes and villains). We could run an ANOVA with an interaction if desired, but we would still — in that case — want to follow up with simple effect tests to test the same pairwise comparisons that we currently present.

*5. Similarly in Study 2: it is not clear whether the authors expected between- or within-political groups differences. The hypothesis suggests, it is between-groups differences that are expected (p. 14: “We predicted that people’s preferences would depend on their political identity, and in particular that political liberals would prefer individualizing-promoting messages whereas political conservatives would prefer binding-promoting messages.”) but in the analysis, the authors compare the two foundations with each other.*

*Additionally, why do authors run two separate regressions for the individualizing and binding MFQ scores (p. 17) and resolution preferences (p. 18)? Running two separate analyses and then comparing the strength of the correlation seems to be a non-standard procedure. Why did authors choose this approach? Instead, I would expect a regression analysis with an interaction (e.g., using mixed models because of the repeated measures).*

*It is also not clear to me how the comparison between the correlations worked. For example, on p. 18, the authors claim the magnitude of the correlations was larger for the binding than individualizing foundations. Does this analysis provide information about differences in strength of the absolute values of the correlations or does it test whether the two correlations significantly differ from each other? If it is the latter, the conclusions about the importance of individualizing moral foundations across political orientation may not be justified because the correlation coefficients had different directions.*

We had two hypotheses in this study, which we now present more clearly. The first hypothesis concerns the (separate) association between political orientation and each foundation category: in particular, that stronger conservatism will predict less individualizing endorsement and more binding endorsement. The second hypothesis concerns the potential difference in the magnitude of the preceding two correlations. We test this second hypothesis via the cocor procedure (and below we discuss convergent results from regression analyses with an interaction term).

Our cocor analysis compares just the magnitude of the correlations, ignoring their sign. (One could run this analysis to compare correlations with different signs, but we did not do this. Our syntax compared the correlations as if they had the same sign, so that the analysis would reveal whether the magnitudes differed.)

It would indeed be conceptually similar to run this as a mixed-model regression with an interaction term. Given the reviewer’s suggestion, we ran this analysis. To do so, we transformed the original dataset into a new one in which each subject occupied two rows (one for each umbrella category of MFT domains: individualizing and binding), with columns to represent their MFQ endorsement scores and their resolution endorsement scores, as well as a column representing their (standardized) political orientation. Notably, if we ran the regression model at this point, it would undoubtedly reveal a political orientation x foundation category interaction, but this would merely reflect the fact that the correlations between political orientation and the MFT categories have different signs (stronger conservatism associated with less endorsement of individualizing foundations and more endorsement of binding foundations). To avoid this interpretation and to instead be able to assess whether the magnitudes of the correlations differed, we inverted participants’ individualizing ratings (computing these new inverted scores as 7 minus their original score in the case of MFQ endorsement, and 8 minus their original score in the case of resolution endorsement, since the original scores were assessed on a 1 to 6 scale and a 1 to 7 scale, respectively). Thus, higher scores on these new inverted scores represent less endorsement of the individualizing foundations. Stronger conservatism is therefore positively correlated with both binding scores and with inverted individualizing scores; the interaction term in the model now assesses whether these two correlations differ in magnitude. We ran two separate regression models — one for MFQ scores and one for resolution scores — predicting endorsement as a function of (standardized) political orientation, foundation category, and their interaction (again, such that individualizing ratings were inverted), including random intercepts for subjects. We’ve included the syntax for both analyses below, and we now present the results of these analyses in the Supplementary Materials file. Both analyses revealed a significant effect of political orientation (stronger conservatism predicted stronger endorsement of binding foundations and of inverted individualizing foundations) and of foundation type (this effect is not conceptually very meaningful because it compares whether endorsement of binding foundations differs from endorsement of inverted individualizing foundations). Importantly, both analyses revealed a significant political orientation x foundation category interaction, revealing that the correlation between political orientation and binding endorsement was stronger than that between political orientation and (inverted) individualizing endorsement. In other words, as compared to endorsement of binding foundations, there is relatively more agreement in the importance of individualizing foundations across political orientation. These regression analyses therefore lead to precisely the same conclusions as those from our cocor analyses. We believe the cocor analyses are more straightforward and easier for readers to follow, so our preference is to present those instead of these multilevel regressions. If there is a strong sentiment otherwise, we can include these regression models in the primary manuscript instead.

summary(lmer(resol_rating~zPolitical_LibLeft*foundation_num+(1|subj),data=Study2_data_hlm))

summary(lmer(MFQ_rating~zPolitical_LibLeft*foundation_num+(1|subj),data=Study2_data_hlm))

*6. I was wondering what characteristics were predictive of loyalty in Study 1 as the differences between villains and heroes were not significant (and looking at Figure 2, there might have been also within-characters differences for this foundation)? Analysis on pp. 13-14 is presented for the two overarching foundations only.*

We examined each of the 10 characteristics that we report in the “Additional characteristics as predictors of individualizing and binding foundations” as predictors of loyalty scores in particular. We are reluctant to report these results in the paper itself because focusing on each separate foundation would introduce a great deal of additional multiple comparisons (50 tests: 10 characteristics for each of the five separate foundations). But we are happy to present the results here. After applying the Benjamini-Hochberg correction that you helpfully recommended below, we found that only a single one of the 10 characteristics significantly predicted loyalty: non-human characters exhibited more loyalty (*M*=4.44) than did White characters (*M*=3.67), corrected B-H *p*-value <.01. None of the other nine characteristics predicted significant differences in loyalty.

*7. I was surprised that in Study 2 the authors analyzed the binding vs. individualizing foundations without looking into the effects of loyalty, given the above mentioned findings for loyalty.*

In Study 2, we didn’t design our resolutions to isolate specific binding foundations. The binding-promoting resolutions arguably target both loyalty and authority simultaneously (e.g., Travis’ decision in *Old Yeller* to follow his mother’s order and shoot the deer to feed their family, thereby demonstrating both loyalty to family and obedience to authority). But in line with your suggestion, we believe it would indeed be helpful for future research to more directly examine people’s preferences for specific binding resolutions. We have added a paragraph to the General Discussion in which we discuss this idea.

*Other comments*

*- Throughout the text (e.g., p. 5), the authors refer to “critical claim of MFT” or “key claims of MFT”, what are these claims?*

Thank you for pointing out that the original manuscript was not clear enough about this. We are now more clear about this in the Introduction. In particular, the key claim of MFT is that there is a systematic association between political identity and endorsement of the distinct moral foundations (whereby stronger conservatism predicts less endorsement of the individualizing foundations and more endorsement of the binding foundations).

*- Please list all the dimensions that were used to describe movie characters in Study 1 to the section “Characteristics coding scheme” instead of directing the readers to the Appendix,*

We now list all characteristics in that section. Thanks for this suggestion.

*- Bonferroni correction used in Study 1 might be over-conservative and reduce statistical power (e.g., Narum, 2006; Perneger, 1998). Authors may look into other approaches to adjust for multiple testing (e.g., Benjamini-Hochberg procedure, 1995, or other methods),*

*Benjamini, Y., & Hochberg, Y. (1995). Controlling the false discovery rate: a practical and powerful approach to multiple testing. Journal of the Royal Statistical Society: Series B (Methodological), 57(1), 289-300.*

*Narum, S. R. (2006). Beyond Bonferroni: less conservative analyses for conservation genetics. Conservation genetics, 7(5), 783-787.*

*Perneger, T. V. (1998). What's wrong with Bonferroni adjustments. BMJ, 316(7139), 1236-1238.*

Thank you for this suggestion. We now report Benjamini-Hochberg corrected p-values, rather than Bonferroni corrected ones (and consequently, a couple of comparisons that previously weren’t reported as statistically significant now are).

*- I think APA standards ask for 2 decimal numbers when presenting statistics (even descriptive ones),*

*- Also regarding APA standards, I think results for the Cohen’s d should be reported with leading 0 (as the values can go higher than 1).*

*- Please provide details for the statistical analysis for t-tests on p.13 as well as report full findings for the regression analysis on p. 18 (last paragraph). They could be reported in the Supplementary materials.*

We now report all numbers to two decimal places and we include a leading 0 for all Cohen’s d values that are less than 1.

In the Supplementary Materials file, we now provide complete syntax and output for all analyses reported in the manuscript.

*- I was also wondering whether the current findings could be expected in other media? For example, children’s literature or comic books? And relatedly, should we expect different effects in the movies for teenagers or adults? Why children’s movies are especially important?*

We think it’s important to study children’s movies rather than movies designed primarily for teen or adult audiences because childhood is the foundational stage for moral development - it is when children are developing morality most actively and therefore might be most influenced by engaging and impactful stimuli like movies. With that said, we appreciate your invitation to consider potential differences in moral depictions in movies for children vs. adults. We have added a paragraph to the General Discussion in which we explore this idea. In short, we think that many of the same patterns/differences would emerge in movies for adults, but that the differences between heroes and villains would be less exaggerated than they are in children’s movies.

As to the question of whether these findings might extend to children’s literature or comic books, or to other media sources such as video games, television shows, and social media channels (e.g., YouTube), we agree this is an intriguing question and one that might be studied in future research.

**Reviewer 2**

*1. The two studies only vaguely related. They don’t address a single problem; they answer very different questions. In particular, the paper doesn’t contain a common aim or a general problem. The paper doesn’t even have an Introduction section that could explain the connection between the two studies. It seems weird to begin the paper with the literature review without knowing the problem under consideration. Due to the lack of common ground for the two studies the paper can be confusing. For example, the second sentence of the abstract points to political differences - unclear why. The added value of the paper is questionable as well, since it’s not clear what literature it is supposed to contribute to: developmental psychology? political science? media studies? As long as the field, and, more importantly, a single research question is defined, the literature review and a (future) introduction should clearly state what particular problem is being solved by the current paper.*

We have updated the Introduction section substantially to more clearly lay out the goal of the paper. The overarching goal is to examine depictions of morality in children’s media. This is a two-pronged approach, and we therefore examine distinct but related questions in each study. Study 1 examines actual depictions of morality (in the form of heroes’ and villains’ moral characteristics), and Study 2 examines people’s preferences for different moral depictions/messages, particularly as related to their own political identity.

*2. Each study taken separately is underdeveloped (see below). If the authors consider further development of the paper, I’d suggest separating it into two different papers and significantly expanding each study. For instance, both studies would benefit from including them into a rich context of communication research, which addressed similar questions for decades, to mention a few: Grizzard, M., Huang, J., Fitzgerald, K., Ahn, C., & Chu, H. (2018). Sensing heroes and villains: Character-schema and the disposition formation process. Communication research, 45(4), 479-501; Grizzard, M., Fitzgerald, K., Francemone, C. J., Ahn, C., Huang, J., Walton, J., ... & Eden, A. (2020). Validating the extended character morality questionnaire. Media Psychology, 23(1), 107-130. Matthews, N. L. (2019). Detecting the boundaries of disposition bias on moral judgments of media characters’ behaviors using social judgment theory. Journal of Communication, 69(4), 418-441. Hastings, A. W. (1993). Moral Simplification in Disney's The Little Mermaid. The Lion and the Unicorn, 17(1), 83-92. Padilla-Walker, L. M., Coyne, S. M., Fraser, A. M., & Stockdale, L. A. (2013). Is Disney the nicest place on earth? A content analysis of prosocial behavior in animated Disney films. Journal of Communication, 63(2), 393-412.*

Thank you for these helpful citations. We now cite several of these papers (Grizzard et al., 2018 and Grizzard et al., 2020 in the Introduction; Hastings, 1993 in the General Discussion) to better situate our study and findings within the literature.

*3. Both studies are presented (until conclusion) in a U.S.-centered manner, including movies, participants, political culture etc. It’s worth mentioning the kind of participants in Study 2 (U.S. adults) and the sample of movies (American) in Study 1. The fact that movies studied were all U.S.-produced needs emphasis, as in other cultures the results would likely differ (e.g. consider Indian cinematography where villains are likely to be individualists, whereas heroes might stick to bonding foundations). The related issue is a representativeness. Both studies use samples and it’s unclear what general population (of movies or persons) they are supposed to represent. In particular, in Study 1:*

*1. How movies were identified as ‘children’s’ rather than ‘general population’s’?*

We identified movies as children’s movies if they: (1) were rated G or PG on IMBD.com, and (2) were rated as appropriate for children 10 years old or younger on commonsensemedia.org. We now include this information in the Method section.

*2. Why the first year of selection was specifically 1948?*

Our primary aim was to select movies from a wide span of years, to ensure that our findings would not apply solely to a given decade or to a narrow range of years. We therefore selected movies spanning a 70-year time period. We chose the late 1940s as the beginning of our date range because the post-World War II period, in part due to the growth of television, saw widespread changes in movie consumption in the United States (Boddy, 1998).

*3. Why top grossing movies were selected rather than e.g. top-rated? What the grossing is supposed to represent?*

We selected top-grossing films because revenue is an indicator of high viewership, which corresponds to more exposure or learning of moral content (whereas high ratings would reflect artistic quality or audience enjoyment). We are interested in how media influences viewers, so revenue was the best objective metric to identify movies with the most widespread influence.

*4. Why movies and not characters were sampled?*

Movies are the entities that the audience consumes (and the entities for which we can determine revenue/viewership), so that was the unit that we sampled. But we are of course ultimately interested in the moral depictions of characters, so once we identified our sample of movies, we coded the characters within them.

*5. Why only (and exactly) seven movies were selected in the end? Are they somehow representative of all the movies.*

*These are important questions as the authors generalize, e.g. ‘These findings reveal that the moral messages conveyed in children’s media…’ should then read ‘These findings reveal that the moral messages conveyed in SEVEN AMERICAN MOVIES…’ and it changes the meaning of conclusions dramatically.*

We now realize that we were not clear enough in the original manuscript. Our complete dataset of characters/movies contained 183 characters from 42 movies. All of our hypothesis testing (and exploratory analyses) were conducted on this complete dataset.

To ensure adequate consistency in the use of the coding scheme, we examined inter-rater reliability using a subset of characters/movies (41 characters from seven randomly selected movies). The only analyses performed on this subset where those used to assess inter-rater reliability (Kappa and ICC).

*In Study 2, the participants come from a convenience sample, therefore all the conclusions should be bound to these specific people rather than to any other population. The results should not be generalized to ‘people in general’ (p.21) or ‘liberals’ or ‘conservatives’ (p.22) in general, because the sample is not representative of any of these groups. The conclusion connects these results to parenting, but the sample doesn’t seem to represent parents as well. What was a median age of the participants?*

Our sample, like almost any other within psychology, was not perfectly representative of the US population. However, the composition of our sample is fairly consistent with the composition of online samples, including those recruited via MTurk (Buhrmester, Kwan, & Gosling, 2011; Paolacci & Chandler, 2014). In addition, as we describe earlier in this document in response to Reviewer 1, Study 2 was sufficiently powered: given our sample size of *N*=110, we had 96% power to detect a correlation of *r*=.35 (which is the minimum correlation between political ideology and foundation endorsement as reported by Graham et al., 2011). The average age of participants in the study was 32.14 years old (*SD* = 15.68). Given the size and composition of our sample, we therefore believe that our conclusions about the relationship between political ideology and moral foundations are justified.

** Throughout the paper the moral foundations are equated with moral values (and ‘relevance’ with ‘importance’), which are not exactly synonyms. ‘Scores of 5 indicated a character clearly and consistently valued the given foundation’, whereas MFQ (Graham et al, 2011) includes ‘relevance’ and ‘agreement’, not importance . This is one issue that makes a difference between foundations which a composite construct (syndrome of social norms, attitudes, beliefs, of which values are only a small part) and values which is much more specific construct (see e.g. Schwartz, S. H. (1992). Universals in the content and structure of values: Theoretical advances and empirical tests in 20 countries. Advances in experimental social psychology, 25(1), 1-65.)*

Thank you for pointing this out. We agree that there can be variability in researchers’ use of constructs like “foundations”, “values”, and other related terms. We have now replaced the term “values” with less ambiguous terms (like “concerns” or “foundations”) in some places throughout the manuscript.

We do still use the term “values” in some places. We believe that our treatment of “foundations” and “values” as more or less interchangeable terms is consistent with the use of these terms in Moral Foundations Theory. Graham et al. (2009) situate their goal of identifying the set of moral foundations as conceptually akin to Schwartz’s (1992) goal of identifying moral values. Graham et al. (2009, p. 1030) write:

“Several theorists have attempted to reduce the panoply of human values to a manageable set of constructs or dimensions. The two most prominent values researchers (Rokeach, 1973; Schwartz, 1992) measured a wide variety of possible values and aggregated them through factor analysis to define a smaller set of core values. Schwartz (1992) and Rokeach (1973) both justified their lists by pointing to the fundamental social and biological needs of human beings.

Moral foundations theory (Haidt & Graham, 2007; Haidt & Joseph, 2004) also tries to reduce the panoply of values, but with a different strategy: We began not by measuring moral values and factor analyzing them but by searching for the best links between anthropological and evolutionary accounts of morality.”

We also note that in most instances where we refer to “value(s)” in the manuscript, we are using the verb form (e.g., to assert that characters endorse or exemplify a particular moral concern).

** Footnote 2: I’d disagree that coders’ guessing what ‘the film clearly intended’ is able to remove personal bias. What would, indeed, remove personal bias is an explicit characteristics given to a character unequivocally by the other characters of the film. Overall, authors’ intention is an outdated construct disproved as early as in the 1960s (e.g. by Roland Barthe).*

Thank you for highlighting this concern. We agree that coding the film’s intended portrayal does not guarantee the elimination of personal bias. We have removed this phrasing from the manuscript.

The only characteristics that relied on this “film’s intention” approach were those that were exploratory and not tied to our central hypotheses (in particular, the character’s attractiveness and weight, which were a subset of the “additional characteristics” that we coded).

** Given there were only two raters, it would be beneficial to mention their gender, race, and nationality. Is there any evidence that children’s perceptions are compatible with those made by the two raters?*

Both coders were White American females.

Evidence from Hamlin and colleagues (e.g., Hamlin et al., 2007; Hamlin & Wynn, 2011) shows that even young infants draw reliable inferences about the prosocial and antisocial nature of characters based on the characters’ behavior in a social interaction. We therefore believe that children would make compatible perceptions of characters’ moral behavior within movies.

** The coding scheme is ambiguous sometimes. For example, Care/Harm includes ‘Is sensitive to the emotions of others’ vs ‘Indifference to the suffering’ whereas many villains are sadistic, that is, they’re not indifferent rather they enjoy suffering of others.*

We took extreme efforts to remove ambiguity from the coding scheme and were able to achieve strong inter-rater reliability, so we believe the coding yielded valid and meaningful objective data. As to this particular point about Care/Harm, we agree that villains are often sadistic, and our coding scheme actually reflected this (e.g., it included “is cruel” and “intentionally harms others” as instances that would justify a code of 1).

** The data provided do not contain the raw scores given by each rater, rather it seems to contain single rater’s responses. It makes, e.g. inter-rater reliability test non-replicable. The ‘movie’ variable is empty.*

We have added a new dataset to the data.Rdata file on the OSF site, which includes the data used to assess inter-rater reliability (i.e., the subset of movies/characters that both coders coded). We have also included the corresponding inter-rater reliability analyses within the analysis code.R file.

We apologize that the ‘movie’ variable was empty in the original data file. We have updated the file and it now includes these data.

** One issue in Study 2 can be related to the use of existing movie stories as stimuli. The familiarity with the movie or a character in general likely affected responses.*

We agree and we considered this potential concern carefully. To address it, we decided to exclude participants’ ratings about movies they had already seen (p. 18 and p. 20).

** What makes a difference between heroes and villains in general? Perhaps their morals is what defines this distinction and not the other way around. That is, it might be that it’s not binding foundation is presented negatively, but it is the very thing that defines a villain.*

Thank you for raising this potential interpretation. We agree that this is certainly possible, and perhaps likely. We have added a paragraph to the General Discussion in which we discuss this idea.
